# Genomic Characterization of Methanomicrobiales Reveals Three Classes of Methanogens

**DOI:** 10.1371/journal.pone.0005797

**Published:** 2009-06-04

**Authors:** Iain Anderson, Luke E. Ulrich, Boguslaw Lupa, Dwi Susanti, Iris Porat, Sean D. Hooper, Athanasios Lykidis, Magdalena Sieprawska-Lupa, Lakshmi Dharmarajan, Eugene Goltsman, Alla Lapidus, Elizabeth Saunders, Cliff Han, Miriam Land, Susan Lucas, Biswarup Mukhopadhyay, William B. Whitman, Carl Woese, James Bristow, Nikos Kyrpides

**Affiliations:** 1 Joint Genome Institute, Walnut Creek, California, United States of America; 2 Joint Institute for Computational Sciences, University of Tennessee – Oak Ridge National Laboratory, Oak Ridge, Tennessee, United States of America; 3 Department of Microbiology, University of Georgia, Athens, Georgia, United States of America; 4 Virginia Bioinformatics Institute, Bioinformatics and Computational Biology Graduate Program, Virginia Polytechnic Institute and State University, Blacksburg, Virginia, United States of America; 5 Department of Biochemistry, Bioinformatics and Computational Biology Graduate Program, Virginia Polytechnic Institute and State University, Blacksburg, Virginia, United States of America; 6 Department of Biological Sciences, Bioinformatics and Computational Biology Graduate Program, Virginia Polytechnic Institute and State University, Blacksburg, Virginia, United States of America; 7 Department of Genetics, Bioinformatics and Computational Biology Graduate Program, Virginia Polytechnic Institute and State University, Blacksburg, Virginia, United States of America; 8 Joint Genome Institute, Los Alamos National Laboratory, Bioscience Division, Los Alamos, New Mexico, United States of America; 9 Bioscience Division, Oak Ridge National Laboratory, Oak Ridge, Tennessee, United States of America; 10 Department of Microbiology, University of Illinois, Urbana, Illinois, United States of America; University of Hyderabad, India

## Abstract

**Background:**

Methanomicrobiales is the least studied order of methanogens. While these organisms appear to be more closely related to the Methanosarcinales in ribosomal-based phylogenetic analyses, they are metabolically more similar to Class I methanogens.

**Methodology/Principal Findings:**

In order to improve our understanding of this lineage, we have completely sequenced the genomes of two members of this order, *Methanocorpusculum labreanum* Z and *Methanoculleus marisnigri* JR1, and compared them with the genome of a third, *Methanospirillum hungatei* JF-1. Similar to Class I methanogens, Methanomicrobiales use a partial reductive citric acid cycle for 2-oxoglutarate biosynthesis, and they have the Eha energy-converting hydrogenase. In common with Methanosarcinales, Methanomicrobiales possess the Ech hydrogenase and at least some of them may couple formylmethanofuran formation and heterodisulfide reduction to transmembrane ion gradients. Uniquely, *M. labreanum* and *M. hungatei* contain hydrogenases similar to the *Pyrococcus furiosus* Mbh hydrogenase, and all three Methanomicrobiales have anti-sigma factor and anti-anti-sigma factor regulatory proteins not found in other methanogens. Phylogenetic analysis based on seven core proteins of methanogenesis and cofactor biosynthesis places the Methanomicrobiales equidistant from Class I methanogens and Methanosarcinales.

**Conclusions/Significance:**

Our results indicate that Methanomicrobiales, rather than being similar to Class I methanogens or Methanomicrobiales, share some features of both and have some unique properties. We find that there are three distinct classes of methanogens: the Class I methanogens, the Methanomicrobiales (Class II), and the Methanosarcinales (Class III).

## Introduction

The Archaea were discovered to form a distinct domain in 1977 [1] and subsequently were found to be comprised of two major kingdoms, the Crenarchaeota and the Euryarchaeota [2]. The Crenarchaeota consist mainly of thermophiles and thermoacidophiles while the Euryarchaeota contains a wider variety of organisms including the methanogens, the extreme halophiles, thermophiles, and thermoacidophiles. Recently a third kingdom, Thaumarchaeota, has been proposed that includes mesophilic organisms previously classified as Crenarchaeota [3].

Methanogens play a major role in the global carbon cycle [4] by carrying out the final steps in the anaerobic degradation of organic material. In the process, they are estimated to produce close to 400 million metric tons of methane per year. Much of the methane is converted back to carbon dioxide by methanotrophs, but some is released to the atmosphere where it is a potent greenhouse gas. As a result of human activities, the concentration of methane in the atmosphere has almost tripled in the last 200 years [5].

Methanogens are currently classified in five orders: Methanobacteriales, Methanococcales, Methanopyrales, Methanomicrobiales, and Methanosarcinales.

It has been recognized that the methanogens can be divided into two major groups based on phylogenetic analysis [6], [7]. The first group contains the orders Methanobacteriales, Methanococcales, and Methanopyrales, and has been named Class I methanogens by Bapteste et al. [7]. The second group, the Class II methanogens, includes Methanomicrobiales and Methanosarcinales. However, the Methanomicrobiales are physiologically more similar to the Class I methanogens than to the Methanosarcinales, growing on H_2_/CO_2_ or formate, while members of the Methanosarcinales can produce methane from acetate, methanol, methylamines, and other C-1 compounds. Recently Thauer et al. [8] have argued that methanogens can be divided into two groups based on the presence or lack of cytochromes, with Methanosarcinales alone possessing cytochromes. The Methanomicrobiales thus belong to the phylogenetic group of Class II methanogens, but to the physiological group of methanogens without cytochromes.

Members of the order Methanomicrobiales have few known unique properties. However their membrane lipid composition is distinctive, and they are unique in possessing aminopentanetetrols in their lipids (reviewed in [9]). In addition to growth on H_2_/CO_2_ or formate, some are capable of using secondary alcohols as electron donors [10]. Methanomicrobiales have been detected in marine environments, in landfills and wastewater reactors, and as symbionts of ciliates (reviewed in [9]).

This is the first publication to describe genomes from the order Methanomicrobiales.

We report here the genome of the marine methanogen *Methanoculleus marisnigri* JR1 [11] and that of *Methanocorpusculum labreanum* Z, a methanogen isolated from tar pit sediments [12]. We include comparisons of these two with the genome sequence of *Methanospirillum hungatei* JF-1, a spiral-shaped methanogen isolated from sewage sludge [13]. We also present a comparative analysis of Methanomicrobiales genomes with those of Methanosarcinales and Class I methanogens.

## Results

### General features

The genomes of *M. labreanum* and *M. marisnigri* consist of one chromosome and no plasmids (Table 1), and the same is true for *M. hungatei*. The size of the *M. hungatei* genome is substantially larger than those of the other two. *M. marisnigri* has only one ribosomal RNA (rRNA) operon, while *M. labreanum* has three and *M. hungatei* has four. In two of the *M. hungatei* rRNA operons, there are two copies of the 5S rRNA.

**Table 1:** General genome statistics.

	*M. labreanum*	*M. marisnigri*	*M. hungatei*
Genome size (bp)	1,804,962	2,478,101	3,544,738
G+C content (bp)	902,600 (50.0%)	1,537,981 (62.1%)	1,600,415 (45.1%)
Number of genes	1828	2559	3305
RNA genes	63 (3.4%)	53 (2.1%)	66 (2.0%)
Protein-coding genes	1765 (96.6%)	2506 (97.9%)	3239 (98.0%)
Pseudogenes	26 (1.4%)	17 (0.7%)	99 (3.0%)
Genes in ortholog clusters	1676 (91.7%)	2294 (89.6%)	3031 (91.7%)
Genes assigned to COGs	1358 (74.3%)	1832 (71.6%)	2314 (70.0%)
Genes assigned to Pfam domains	1335 (73.0%)	1790 (69.9%)	2326 (70.4%)
Genes with signal peptides	406 (22.2%)	620 (24.2%)	771 (23.3%)
Genes with transmembrane helices	368 (20.1%)	595 (23.3%)	762 (23.1%)
Fusion genes	73 (4.0%)	104 (4.1%)	171 (5.2%)
Transposable elements	0	3	76
CRISPR-associated genes	8	1	21
CRISPR repeat arrays	1	0	6

### Methanogenesis

As expected, the three Methanomicrobiales have all of the genes required for methanogenesis from hydrogen and carbon dioxide. All three species are capable of utilizing formate, and they have formate transporters as well as cytosolic formate dehydrogenases that probably reduce coenzyme F_420_. No homologs were found to C-1 compound:corrinoid methyltransferases, corrinoid proteins, and methylcobalamin:Coenzyme M methyltransferases involved in methanogenesis from methanol and methylamines (no BLAST hit to *Methanosarcina acetivorans* proteins with 10^−5^ cutoff value). Some methanogens, including *M. marisnigri*, can utilize secondary alcohols as electron donors for methanogenesis [10], whereas *M. hungatei* JF-1 can not [14], and *M. labreanum* has not been tested. Alcohol dehydrogenases that oxidize secondary alcohols and use the electrons to reduce coenzyme F_420_ have been characterized [15] and the structure has been determined for one enzyme [16]. *M. marisnigri* has a gene (Memar_0783) that is closely related to the F_420_-dependent secondary alcohol dehydrogenase from *Methanoculleus thermophilus*, but *M. labreanum* and *M. hungatei* do not (no BLAST hit with cutoff of 10^−10^).

**Figure 1:**
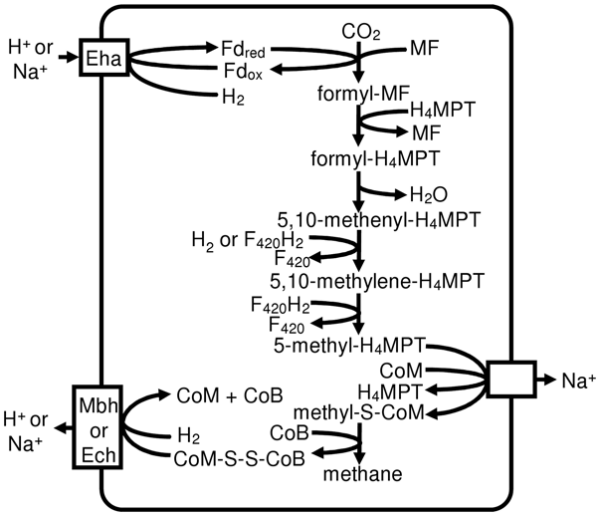
Proposed pathway for methanogenesis in Methanomicrobiales. Methanomicrobiales are predicted to couple formylmethanofuran formation and CoM-CoB heterodisulfide reduction to ion gradients. Fd: ferredoxin; MF: methanofuran; H_4_MPT: tetrahydromethanopterin.

In Class I methanogens, F_420_-non-reducing hydrogenase provides electrons to heterodisulfide reductase, and its D subunit interfaces with heterodisulfide reductase [17]. In all three Methanomicrobiales the gene for the D subunit of the hydrogenase (Mlab_0242, Memar_0622, Mhun_1839) is adjacent to the genes for heterodisulfide reductase, but phylogenetic analysis of hydrogenase alpha subunits (not shown) reveals that only *M. marisnigri* possesses the F_420_-non-reducing hydrogenase (Memar_1007–1008) (Table 2). Apparently, *M. labreanum* and *M. hungatei* use a different source of electrons for their heterodisulfide reductase. Based on the lack of F_420_-nonreducing hydrogenase and the fact that the Eha hydrogenase is located adjacent to formylmethanofuran dehydrogenase (Fmd) (see below), we propose that, in at least some Methanomicrobiales, Fmd and heterodisulfide reductase are linked to transmembrane proton or sodium ion transport (Figure 1) rather than flavin-based electron bifurcation as proposed by Thauer et al. [8].

**Table 2:** Hydrogenases in methanogen genomes.

	Frh	Mvh	Eha	Ehb	Ech	Mbh
Class I methanogens	All	All	all except Msp	all except Mka		
Methanosarcinales	Mac, Mba, Mmz				Mba, Mmz	
Methanomicrobiales	All	Mmar	All		All	Mlab, Mhun

Frh: F_420_-reducing hydrogenase; Mvh: F_420_-non-reducing hydrogenase; Eha: energy-converting hydrogenase A; Ehb: energy-converting hydrogenase B; Ech: energy-converting hydrogenase; Mbh: membrane-bound hydrogenase; Msp: *Methanosphaera stadtmanae*; Mka: *Methanopyrus kandleri*; Mac: *Methanosarcina acetivorans*; Mba: *Methanosarcina barkeri*; Mmz: *Methanosarcina mazei*; Mmar: *Methanoculleus marisnigri*; Mlab: *Methanocorpusculum labreanum*; Mhun: *Methanospirillum hungatei*.

*M. labreanum* has a hydrogen-forming methylene-tetrahydromethanopterin dehydrogenase (COG4074), an enzyme previously found only in Class I methanogens. The other two genomes lack genes assigned to COG4074 and thus are unlikely to have this enzyme. The enzyme functions under conditions of nickel limitation (reviewed in [18]), so this suggests that *M. labreanum* can tolerate lower environmental nickel concentrations. When this gene is found in a Class I methanogen genome, it is often accompanied by one or two paralogs of unknown function belonging to COG4007. However, *M. labreanum* lacks these paralogs.

### Membrane-Bound Hydrogenases

Methanogens have several families of membrane-bound hydrogenases that participate in various processes including methanogenesis and biosynthesis (reviewed in [19]). These hydrogenases are encoded by a core of conserved genes that includes from six to more than 20 subunits. The three Methanomicrobiales genomes encode two to three membrane-bound hydrogenases (Table 2). All three possess the genes for a membrane-bound hydrogenase similar to that encoded by the *Methanothermobacter thermautotrophicus eha* operon (Memar_1172–1185, Mlab_0561–0573, Mhun_2094–2106). Their genes for the enzyme subunits are in the same order as those in the *M. thermautotrophicus eha* operon. However, some of the smaller subunits have diverged so extensively that homology can not be detected, and subunits A and M are absent. Adjacent to the hydrogenase operon are genes for the subunits of formylmethanofuran dehydrogenase, suggesting that the Eha hydrogenase may reduce the ferredoxin used by this enzyme (Figure 1).

All three genomes also have a six-subunit membrane-bound hydrogenase operon similar to Ech hydrogenase (Mlab_1619–1624, Memar_0359–0364, Mhun_1741–1746), which has multiple functions in *Methanosarcina barkeri* [20]. *M. labreanum* and *M. hungatei,* but not *M. marisnigri*, also have an operon very similar to the *mbh* operon of *Pyrococcus furiosus*. Since this hydrogenase is found in the two Methanomicrobiales genomes that lack F_420_-nonreducing hydrogenase, the Mbh hydrogenase may be involved in heterodisulfide reduction (Figure 1). *M. hungatei* has another operon similar to membrane-bound hydrogenases (Mhun_1817–1822). Homologous operons are absent from the other two Methanomicrobiales, but they are found in two Methanosarcinales, *Methanosarcina acetivorans* and *Methanosarcina mazei*. However, the hydrogenase large subunits of these operons appear to lack the cysteine residues necessary for binding to the nickel-iron center, so these operons may not encode hydrogenases.

### Metabolism and Transport

The Embden-Meyerhof pathway is present in many methanogens, where it is thought to play a role in the metabolism of stored glycogen. Although *M. hungatei* and *M. marisnigri* have putative glycogen phosphorylases (Mhun_1203, Memar_1262, Memar_2480), and *M. marisnigri* has a putative glycogen branching enzyme (Memar_1265), none of the three Methanomicrobiales has an identifiable glycogen synthase. *M. marisnigri* and *M. hungatei* appear to encode a complete glycolysis pathway. This suggests that they may be able to utilize glucose from the environment (although they lack identifiable sugar transporters) or that they have a novel glycogen synthase. *M. hungatei* was previously reported to lack phosphofructokinase activity [21], but the genome contains two putative phosphofructokinase genes (Mhun_0556 and Mhun_1465). The Embden-Meyerhof pathway appears to be absent from *M. labreanum* as it lacks both phosphofructokinase and pyruvate kinase. A gluconeogenesis pathway is present in all three, as it is necessary for biosynthesis of pentoses and hexoses.

**Figure 2:**
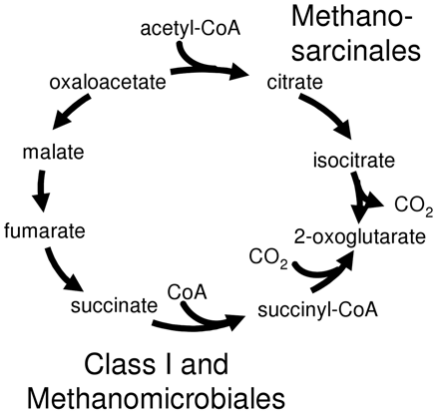
Alternate pathways for synthesis of 2-oxoglutarate from oxaloacetate. Class I methanogens and Methanomicrobiales use a partial reductive citric acid cycle while Methanosarcinales use a partial oxidative citric acid cycle.

The pathway for 2-oxoglutarate production differs significantly between the Methanosarcinales and the Class I methanogens. Methanosarcinales generate 2-oxoglutarate through a partial oxidative TCA cycle with isocitrate as an intermediate, while Class I methanogens use a partial reductive TCA cycle with succinate as an intermediate (Figure 2). Methanomicrobiales appear to use the partial reductive TCA cycle, similar to the Class I methanogens, as they have genes for all of the necessary enzymes and they lack genes for citrate synthase and isocitrate dehydrogenase. They possess genes encoding the two subunits of the predicted archaeal aconitase [22], but this enzymatic activity has not been verified experimentally.

### Sigma Factor Regulators

Both *M. labreanum* and *M. marisnigri* contain an anti-anti-sigma factor (Memar_02467, Mlab_1451), an anti-sigma factor (Memar_2469, Mlab_1452), and a serine phosphatase (Memar_2468, Mlab_1450) that are similar to the SpoIIAA/SpoIIAB/SpoIIE components of the *Bacillus subtilis* sporulation pathway. Moreover, these SpoII-type proteins are also found in *M. hungatei*, but not outside the order Methanomicrobiales. This finding is intriguing given that no *bona fide* sigma factors have been identified in Archaea. Kyrpides and Ouzounis identified proteins in *M. jannaschii* with similarity to conserved region 4 of bacterial sigma factors [23], and the Methanomicrobiales have homologs of three of these proteins (MJ0173, MJ0272, and MJ1243). However, the SpoIIAB anti-sigma factor binds to three separate regions of sigma F [24] corresponding to conserved regions 2, 3, and 4, and regions 2 and 3 are not present in the archaeal proteins. Therefore the targets of these archaeal anti-sigma factors can not be determined from the genome sequence.

### Phylogenetic Analysis of Enzymes for Methanogenesis and Cofactor Biosynthesis

**Figure 3:**
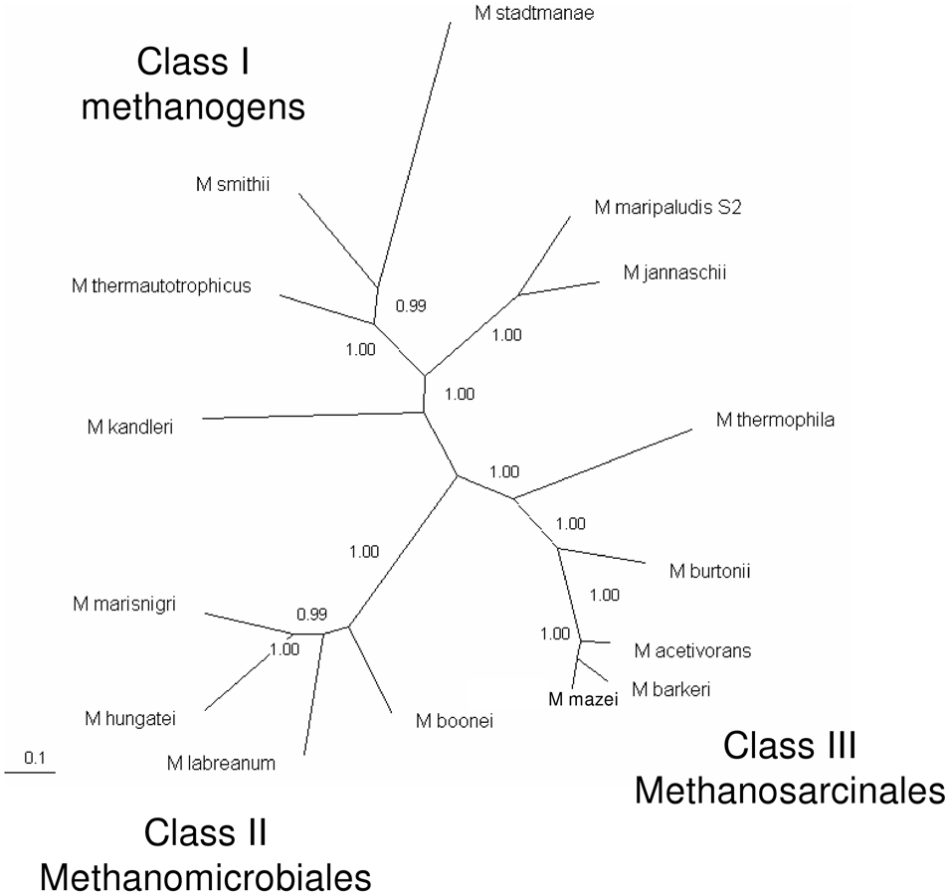
Phylogenetic tree of methanogens based on seven core enzymes of methanogenesis and cofactor biosynthesis. See Materials and Methods for a list of the proteins and organisms included. Protein sequences were concatenated and aligned with Clustal W. The tree was generated with MrBayes 3.1.2 and viewed with TreeView.

Bapteste et al. [7] determined the relationships among the various groups of methanogens by generating phylogenetic trees for enzymes of methanogenesis and cofactor biosynthesis. Their analysis found that methanogens could be divided into two groups: Class I and Class II methanogens. We present here an updated analysis that includes additional sequenced genomes. Furthermore, the protein-coding genes that we used in the analysis (see Materials and Methods) are present in only one copy per genome. Inclusion of the additional genomes reveals that, surprisingly, Methanomicrobiales are equally distant from Class I methanogens and from the Methanosarcinales (Figure 3). Therefore there appear to be three distinct classes of methanogens: the Class I methanogens, the Methanomicrobiales (that we have termed Class II methanogens), and the Methanosarcinales (that we have termed Class III methanogens).

### Comparative genomics of methanogens

Now that several sequenced genomes from the order Methanomicrobiales are available, it is possible to carry out comparative genomic analyses between this order and the other methanogens. We used a protein clustering method to identify and cluster related proteins from 15 species representing Class I, II, and III methanogens (see Materials and Methods for the list of organisms). We then searched for the signature clusters, i.e. clusters of homologous proteins that are present in all members of a phylogenetic group and absent from other groups. Of particular interest are the exclusive signature clusters, those whose member proteins are found in all sequenced genomes from only one class. We also identified shared signature clusters (present in only two classes) and common signature clusters (present in all three).

**Figure 4:**
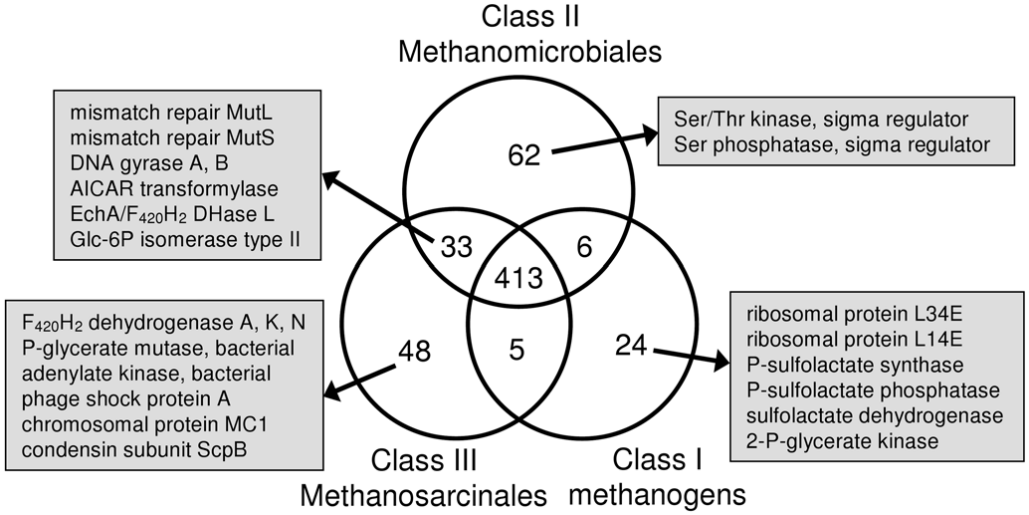
Venn diagram of signature clusters. The clusters were generated using a spectral clustering procedure (see Materials and Methods section for details). Signature protein clusters were identified as clusters for which a member protein was present in every analyzed species from one or more classes of methanogens. The number of exclusive, shared, and common signature clusters associated with each methanogen class are shown. The functions of characterized proteins belonging to exclusive signature clusters and to clusters shared between the Methanomicrobiales and the Methanosarcinales are also noted.

We found 413 common signature clusters (Figure 4, Supplementary Table S1). These proteins are involved primarily in core information processing and essential metabolic activities (i.e. transcription, translation, methanogenesis, etc.). We found 62 exclusive signature clusters for Methanomicrobiales, 24 for Class I methanogens, and 48 for Methanosarcinales. Given the relatively close phylogenetic relationship between Methanomicrobiales and Methanosarcinales in ribosomal RNA and ribosomal protein-based trees, it is surprising that they share only 33 clusters to the exclusion of the Class I methanogens. While this is more than either class shares with the Class I methanogens, it represents but a very small proportion of the genome. In the following sections we describe some of the signature proteins associated with each of the three classes, as well as those shared by Methanomicrobiales and Methanosarcinales.

#### Class I

The Class I methanogen exclusive signature clusters include two LSU ribosomal proteins (L34E and L14E) and three enzymes of coenzyme M (CoM) biosynthesis (phosphosulfolactate synthase, phosphosulfolactate phosphatase, and sulfolactate dehydrogenase). This suggests that other methanogens possess either unrelated genes for these enzymes or a different pathway for CoM biosynthesis. Also present in only Class I methanogens is 2-phosphoglycerate kinase, an enzyme used in the synthesis of cyclic 2,3-diphosphoglycerate, which is thought to be a thermoprotectant. Its presence in mesophilic Class I methanogens suggests that it carries out a different function in these organisms. The second enzyme of the pathway, cyclic 2,3-diphosphoglycerate synthetase, is found only in a subset of Class I methanogens and is not part of the signature.

All Class I methanogens also have a homolog of seryl-tRNA(Sec) selenium transferase, used for the synthesis of selenocysteine in bacteria. However, this gene is likely to have a different function in archaea because not all of the Class I methanogens use selenocysteine [25], and those that do utilize a different pathway for selenocysteine synthesis, one that is shared with eukaryotes [26], [27]. Experimental testing of this protein found that it did not catalyze selenocysteine formation [28].

#### Methanomicrobiales (Class II)

Of the 62 exclusive signature clusters for Methanomicrobiales, 26 are hypothetical proteins, reflecting the fact that this order has been less studied. A serine/threonine kinase and a serine phosphatase, both of which regulate sigma factors in bacteria (see the Sigma Factor Regulators section above), are part of the Methanomicrobiales signature. In addition to a full-length heterodisulfide reductase subunit A (HdrA), Methanomicrobiales also contain a homolog that is truncated at both the N- and C-terminus. Similarly, the A and G subunits of their tetrahydromethanopterin S-methyltransferase are fused. A separate A subunit was found, but no other G subunit is present.

#### Methanosarcinales (Class III)

Among the exclusive signature proteins found in Methanosarcinales are subunits A, K, and N of reduced coenzyme F_420_ (F_420_H_2_) dehydrogenase. Since only Methanosarcinales can use methyl compounds as a substrate for methanogenesis, it is not surprising that this enzyme, used for growth on methyl compounds, is not found in the other methanogens. All methanogens have the archaeal bisphosphoglycerate-independent phosphoglycerate mutase, but Methanosarcinales also have a bacterial version. Similarly, Methanosarcinales use the bacterial adenylate kinase while other methanogens have the archaeal enzyme.

Methanosarcinales exclusive signature proteins include phage shock protein A, a protein that functions in the repair of damaged cell membranes. Likewise, they encode two proteins involved in DNA compaction: the non-histone chromosomal protein MC1 and a unique variant (∼200 amino acids longer) of the ScpB subunit of the condensin complex. That these two chromosome condensation proteins are found only in Methanosarcinales may be related to the larger genome size of some members. The other two components of the condensin complex, ScpA and Smc, are present in most methanogens, including Methanosarcinales. In addition to these DNA condensation proteins, all methanogens have at least one histone gene. Most also have a copy of the gene encoding the Alba protein, but among Methanosarcinales it is present only in *Methanosaeta thermophila*.

#### Missing from Class I

There are 33 clusters shared by Methanomicrobiales and Methanosarcinales that are absent from Class I methanogens. Among these are the DNA mismatch repair proteins MutL and MutS. MutH, however, is not present in any methanogen. This suggests that, if Class I methanogens have methyl-directed mismatch repair, they use a different system. Class I methanogens also lack DNA gyrase subunits A and B. This is unexpected as several Class I methanogens were found to be sensitive to coumarins that target bacterial DNA gyrase [29]. Furthermore, DNA gyrase is the only protein known to introduce negative supercoils into DNA, and these are required for many cellular processes including transcription and DNA replication [30]. Another enzyme missing from Class I methanogens is 5-amino-4-imidazolecarboxamide ribonucleotide (AICAR) transformylase in the pathway for *de novo* purine synthesis. Since most Class I methanogens are autotrophs, they must have this capability provided by a protein unrelated to the known enzyme.

Another shared cluster is the one containing Ech hydrogenase subunit A. Although *M. acetivorans* lacks Ech, it does have the F_420_H_2_ dehydrogenase subunit L and a subunit of multisubunit sodium/proton antiporters, both of which cluster with EchA.

Lastly, Methanomicrobiales and Methanosarcinales have one form of glucose-6-phosphate isomerase (COG2140), while most Class I methanogens use another (COG0166). Since a glucose-6-phosphate isomerase could not be identified in *Methanopyrus kandleri* or in Methanobacteriales, there is probably a third form of this enzyme.

## Discussion

### Phylogenetics

The sequencing of the genomes of *M. labreanum* and *M. marisnigri* reported in this paper, combined with the previously sequenced genome of *M. hungatei*, has enabled further characterization of the order Methanomicrobiales and clarification of its relationship to other methanogens. Our analyses including these species reveal that the order Methanomicrobiales is clearly distinct from other methanogens. The phylogenetic tree built for seven core methanogenesis and cofactor biosynthesis enzymes reveals three discrete groups of methanogens: the Class I methanogens, the Methanomicrobiales (termed here Class II), and the Methanosarcinales (termed here Class III). This classification differs significantly from the previous study by Bapteste et al. [7] that divided the methanogens into two major groups. In that earlier study, the order Methanosarcinales was represented by only species from the genus *Methanosarcina*, whereas our study also included two genomes from other genera. Likewise, their analysis included only one representative of the Methanomicrobiales, while we included four species from this order. Because our study encompassed more species and greater diversity, our results may be a more accurate representation of the relationships among these groups. A relatively close relationship was previously seen between Methanosarcinales and Methanomicrobiales in 16S rRNA trees [3], [31] and ribosomal protein trees [3], [7]. In contrast, Methanomicrobiales are equally distant from Class I methanogens and Methanosarcinales in the tree built in this study from core methanogenesis proteins.

### Genomic Analyses

The protein clustering results reported also suggest a significant distance between Methanomicrobiales and all other methanogens. They share only 6 signature clusters with Class I methanogens and 33 with Methanosarcinales. In addition, the number of exclusive signature clusters for the Methanomicrobiales is of the same magnitude as the signatures for the other two groups. The complement of membrane-bound hydrogenases also shows the uniqueness of Methanomicrobiales. They all have the Eha hydrogenase similar to Class I methanogens and the Ech hydrogenase found in Methanosarcinales, while some of them have hydrogenases similar to Mbh from *P. furiosus* and a putative membrane-bound hydrogenase from Methanosarcinales.

Methanomicrobiales share some capabilities with Class I methanogens to the exclusion of Methanosarcinales. Both groups are capable of using only H_2_/CO_2_ or formate for methanogenesis. The genomes show that they also share the pathway for 2-oxoglutarate synthesis. Both use a partial reductive TCA cycle, while Methanosarcinales use a partial oxidative TCA cycle. This could reflect the observations that Methanomicrobiales efficiently use low concentrations of H_2_, while the Methanosarcinales dominate in environments in which acetate is plentiful. The partial oxidative TCA cycle results in the loss of one carbon as CO_2_, therefore the use of the reductive cycle by Methanomicrobiales and Class I methanogens would be predicted to preserve more fixed carbon. On the other hand, we propose that, similar to Methanosarcinales, Methanomicrobiales link formylmethanofuran synthesis and heterodisulfide reduction to membrane ion gradients, even though they lack cytochromes and methanophenazine that are present in Methanosarcinales.

### Hydrogenases

Methanomicrobiales encode from two to four membrane-bound hydrogenases. In all three genomes (*M. labreanum*, *M. marisnigri*, and *M. hungatei*), the genes for Eha hydrogenase are found adjacent to genes for formylmethanofuran dehydrogenase (Fmd), suggesting that the Eha hydrogenase may reduce a low potential ferredoxin that is required for the reduction of CO_2_ to formylmethanofuran. In contrast, in the Class I methanogen *Methanococcus maripaludis*, the *eha* and *fmd* operons are not linked, and Eha hydrogenase presumably plays a role in carbon assimilation similar to Ehb and not methanogenesis [8], [32], [33].

All Methanomicrobiales also contain genes for the Ech hydrogenase that has been characterized in *M. barkeri*. Ech hydrogenase is involved in reduction of ferredoxin for the first step of methanogenesis from H_2_/CO_2_, in the reduction of ferredoxin for biosynthesis, and in the formation of H_2_ from ferredoxin during aceticlastic methanogenesis [20]. Since the Ech hydrogenase is found in all three Methanomicrobiales, it is likely that its function is common to all three, e.g. the reduction of ferredoxin for 2-oxoglutarate synthesis. Another putative membrane-bound hydrogenase (Pmh) is found only in *M. hungatei* where it may perform a function that is unique to this organism, such as producing ferredoxin for acetyl-CoA decarbonylase/synthase, an enzyme that is absent from the other two.

Experimental evidence is needed to determine the functions of these hydrogenases. Nevertheless, their distribution within the Methanomicrobiales is clearly distinct from that in the Class I methanogens and the Methanosarcinales (Table 2), supporting the functional and evolutionary uniqueness of this group. Their distribution and other features of the operons suggest that their roles in energy conservation differ in Class I methanogens, Methanosarcinales, and Methanomicrobiales.

## Materials and Methods

### DNA Preparation

*M. marisnigri* strain JR1 was obtained from the ATCC (ATCC 35101). It was cultured at room temperature in modified McC medium [34] that contained 0.1 M NaCl, 3 g/L of sodium bicarbonate, 2 g/L of Trypticase (replacing yeast extract), and 0.17 g/L of Na_2_S·9H_2_O. *M. labreanum* strain Z was obtained from the ATCC (ATCC 43576). It was cultured at 37°C in MS-OCM Base Medium with 2.5 g/L NaCl, 5 mM sodium acetate, 50 mM sodium formate, and 2.5% (v/v) of rumen fluid.

For DNA isolation, cells were suspended in TE buffer (10 mM Tris, 1 mM EDTA, pH 8.0). Sodium dodecyl sulfate was added to a final concentration of 0.5% and proteinase K was added to make 100 micrograms/ml, then the solution was incubated at 37°C for 1 hour. After adding NaCl to 0.5 M concentration, the solution was approximately 0.9 ml. Next, 0.5 ml chloroform:isopropyl alcohol (24∶1) was added. The solution was mixed and then centrifuged at 13,000×g for 10 minutes. The aqueous phase was transferred to a new tube, combined with 0.5 ml phenol:chloroform:isoamyl alcohol (25∶24∶1), mixed, and centrifuged at 13,000×g for 10 minutes. The aqueous phase was collected, combined with 0.6 ml isopropanol, incubated at room temperature for 30 minutes, and then centrifuged at 13,000×g for 5 minutes. The pellet was washed with 70% ethanol, resuspended in TE+RNAse (100 micrograms/ml), and incubated at 37°C for 20 minutes.

### Genome Sequencing and Assembly

The genome of *M. labreanum* Z was sequenced at the Joint Genome Institute (JGI) using a combination of Sanger shotgun sequencing and 454 sequencing-by-synthesis technology. All general aspects of library construction and sequencing performed at the JGI can be found at http://www.jgi.doe.gov/sequencing/protocols/prots_production.html. Draft assemblies were based on 26,432 Sanger shotgun and 390,106 pyrosequencing reads. The combined reads provided 34× coverage of the genome. The Newbler assembly software (www.454.com) and the Paracel Genome Assembler (Paracel, Pasadena, CA) were used for fragment assembly, and the Consed finishing package (www.phrap.org) was used for quality assessment and editing. All mis-assemblies were corrected and all gaps between contigs were closed by custom primer walk using subclones or PCR products as templates. A total of 196 additional reactions were run to close gaps and to raise the quality of the finished sequence.

The genome of *M. marisnigri* JR1 was sequenced at the Joint Genome Institute (JGI) using a combination of 3 kb, 7 kb and 36 kb (fosmid) DNA libraries. Draft assemblies were based on 29,769 total reads. The three libraries combined provided 11× coverage of the genome. The Phred/Phrap/Consed software package (www.phrap.com) was used for sequence assembly and quality assessment [35]–[37]. All mis-assemblies were corrected and all gaps between contigs were closed by custom primer walk using subclones or PCR products as templates. A total of 702 primer walk reactions, PCR end reads and 3 mini-libraries were required to close gaps and to raise the quality of the finished sequence.

### Genome Analysis

Automatic genome annotation was performed at Oak Ridge National Laboratory. Genes were identified using a combination of Critica [38] and Glimmer [39]. In addition, predicted coding regions (CDSs) were manually curated using JGI's Gene-PRIMP Quality Assurance pipeline (http://tunis.jgi-psf.org/geneprimp) (Pati et al., in preparation). Comparative genome analysis was performed within the Integrated Microbial Genomes (IMG) system [40]. CRISPR repeats were identified with the CRISPR Recognition Tool [41].

A phylogenetic tree was constructed using the concatenated sequences of seven core proteins found in all methanogens and involved in methanogenesis and cofactor biosynthesis. The genes included are F_420_-dependent methylenetetrahydromethanopterin dehydrogenase (*mtd*, COG1927), tetrahydromethanopterin:coenzyme M methyltransferase subunits B (*mtrB*, COG4062), C (*mtrC*, COG4061), D (*mtrD*, COG4060) and E (*mtrE*, COG4059), F_O_ synthase subunit 1 (*cofG*), and sulfopyruvate decarboxylase alpha subunit (*comD*). Protein sequences were downloaded from IMG [40]. The concatenated amino acid sequences were aligned with Clustal W [42], and the tree was generated with MrBayes 3.1.2 [43] with 1,000,000 generations sampled every 100 generations. The first 250,000 generations were discarded as burn-in. The tree was visualized with TreeView [44].

For protein clustering, methanogens were included from all three groups: six Class I methanogens, four Methanomicrobiales, and five Methanosarcinales. Class I included *Methanocaldococcus jannaschii*, *Methanothermobacter thermautotrophicus*, *Methanopyrus kandleri*, *Methanococcus maripaludis* S2, *Methanobrevibacter smithii*, and *Methanosphaera stadtmanae*. Methanomicrobiales included *Methanocorpusculum labreanum*, *Methanoculleus marisnigri*, *Methanospirillum hungatei*, and Candidatus *Methanoregula boonei*. Methanosarcinales included *Methanosarcina acetivorans*, *Methanosarcina mazei*, *Methanosarcina barkeri*, *Methanococcoides burtonii*, and *Methanosaeta thermophila*. Protein sequences for these organisms were downloaded from IMG [40]. We applied a spectral clustering procedure [45], [46] to cluster similar proteins based on the topology of their similarity graph, rather than using a fixed threshold value for sequence similarity. The proteins are represented as nodes in a connected undirected graph with edges that carry weights based on node-to-node similarity according to the protein identity. The clustering procedure is analogous to a random walk of a particle moving on this graph from one node to another. In each node the particle moves to another node based on the probabilities corresponding to the weights of the edges. The amount of time the particle spends in a given subgraph will determine whether this is indeed a cluster of its own or not.

The second eigenvalue of the transition matrix is a measure of how easily a graph (i.e. a cluster) can be partitioned. A cutoff value of 0.8 was applied; if the second eigenvalue exceeds 0.8, the cluster is further partitioned. This approach provides a relatively flexible partitioning that can reveal protein similarities despite sequence differences due to phylogenetic distance.

Signature protein clusters were identified as clusters for which a member protein was present in every analyzed species from one (or more) class of methanogens. Those clusters were binned into groups: exclusive signature clusters found in all members of only one class, shared signature clusters found in all members of a specified pair of classes, and common clusters found in all three classes. The resultant cluster distribution was visualized as a Venn diagram.

### Accession Numbers

The genome sequences of *Methanoculleus marisnigri* JR1, *Methanocorpusculum labreanum* Z, and *Methanospirillum hungatei* JF-1 can be accessed in GenBank (CP000562, CP000559, and CP000254, respectively). The Genomes OnLine Database accession numbers are Gc00512, Gc00506, and Gc00350, respectively.

## Supporting Information

Table S1 Signature clusters of methanogens. List of signature clusters including exclusive clusters that are present in one class only, shared clusters that are present in two classes, and common clusters that are present in all three classes.
(0.11 MB TXT)
